# Naringenin and cryptotanshinone shift the immune response towards Th1 and modulate T regulatory cells via JAK2/STAT3 pathway in breast cancer

**DOI:** 10.1186/s12906-022-03625-x

**Published:** 2022-05-23

**Authors:** Shokoofe Noori, Mitra Nourbakhsh, Hossein Imani, Niloofar Deravi, Niloufar Salehi, Zohreh Abdolvahabi

**Affiliations:** 1 Department of Biochemistry, Faculty of Medicine, Shahid Beheshti University of Medical Sciences, Tehran, Iran; 2 Finetech in Medicine Research Center, Iran University of Medical Sciences, Tehran, Iran; 3 Department of Biochemistry, Faculty of Medicine, Iran University of Medical Sciences, Tehran, Iran; 4 Nutrition Department, School of Nutritional Sciences and Dietetics, Tehran University of Medical Sciences, Tehran, Iran; 5 Metabolic Diseases Research Center, Research Institute for Prevention of Non-Communicable Diseases, Qazvin University of Medical Sciences, Qazvin, Iran

**Keywords:** Naringenin, Cryptotanshinone, Immunomodulator, Regulatory T cells, STAT3, Breast cancer

## Abstract

**Background:**

Use of natural products has been proposed as an efficient method in modulation of immune system and treatment of cancers. The aim of this study was to investigate the potential of cryptotanshinone (CPT), naringenin, and their combination in modulating the immune response towards Th1 cells and the involvement of JAK2/STAT3 signaling pathway in these effects.

**Methods:**

Mouse models of delayed type hypersensitivity (DTH) were produced and treated with naringenin and CPT. The proliferation of spleen cells were assessed by Bromodeoxyuridine (BrdU) assay. Flowcytometry and enzyme-linked immunosorbent assay (ELISA) tests were employed to evaluate subpopulation of T-lymphocytes and the levels of cytokines, respectively. The JAK/STAT signaling pathway was analyzed by Western blotting.

**Results:**

We showed higher DTH, increased lymphocyte proliferation, decreased tumor growth and reduced JAK2/STAT3 phosphorylation in mice treated with naringenin and CPT. Moreover, a significant decline in the production of IL-4 and an upsurge in the production of IFN-γ by splenocytes were observed. Additionally, the population of intra-tumor CD4^+^CD25^+^Foxp3^+^ T cells was significantly lower in naringenin + CPT treated animals than that in controls.

**Conclusion:**

Naringenin-CPT combination could exert immunomodulatory effects, suggesting this combination as a novel complementary therapeutic regimen for breast cancer.

**Supplementary Information:**

The online version contains supplementary material available at 10.1186/s12906-022-03625-x.

## Introduction

Cancer is the leading cause of death in the world and its incidence and mortality are rapidly growing [1]. Globally, breast cancer is the main cause of cancer-related deaths in females [2, 3]. Without changes in the current therapeutic systems, the number of females diagnosed with breast cancer would approximately double to 3.2 million by 2030 [3]. Primarily, surgical resection, chemotherapy, and fractionated radiotherapy are used to treat cancer. However, the effectiveness of some therapies is constrained by treatment-associated side effects, drug resistance, and off-target effects. Furthermore, conventional treatments do not typically eliminate metastatic cancer cells; so, there is a high probability of recurrence [4]. Therefore, more effective adjuvant therapies may reduce the burden of this disease.

Natural and herbal products are alternatives for prevention and adjuvant therapy of cancer with fewer side effects [5]. Naringenin, a member of the flavanone family, is abundantly present in citrus fruits. Recent studies have shown that naringenin has anti-carcinogenic, anti-oxidant, anti-atherogenic, and potential immune-modulating activities [6].

A traditional Chinese herb, known as Salvia Miltiorrhiza Bunge (Danshen), has been widely applied for the treatment of many diseases, such as diabetes, ischemic stroke, hepatitis, chronic renal failure, menstrual disorders, osteoporosis, and cardiovascular diseases [7–9]. One of the typical effective substances isolated from Danshen, known as Cryptotanshinone (CPT), has been revealed to have various pharmacological activities, including anti-angiogenic, anti-inflammatory, anti-oxidative, anti-diabetic, and anti-obesity properties. In several cancer cells, such as breast cancer, hepatocarcinoma, prostate carcinoma, rhabdomyosarcoma, as well as melanoma cells, CPT has been shown to have noticeable anti-tumor activity [5, 10, 11]. Immunomodulatory effects have also been proposed for CPT [12]; suggesting that this substance could influence the response of cancer cells to immunotherapy.

Cancer immunotherapy, the immune system’s artificial stimulation, is an efficient method for the treatment of different cancers [13]. Immunotherapy engages the immune system in the battle against cancer by taking advantage of the capacity of the T lymphocyte for antigen-directed cytotoxicity [14]. CD4 T helper (Th) cells are characteristically classified into five main subcategories including, Th1, Th2, Th17, Tfh and Treg (T regulatory) [15]. Th1 cells secrete interferons, while Th2 cells are known for being able to secrete interleukin 13 (IL-13), IL-10, IL-5, and IL-4 production.

The development of T cells depends on their microenvironment, with cytokines playing a major role in this procedure. IFN γ/IL-12 drives Th1, and IL-4 promotes Th2 polarization [16]. Since Th1 pathways typically lead to the activation of natural killer (NK) cells, cytotoxic T-cell lymphocytes (CTL), monocytes, and macrophages, shift of immune response towards Th1 leads to tumor rejection [17].

Evidence shows that there is a state of chronic inflammation at tumor sites and that IL-4, particularly, is upregulated in the tumor microenvironment. Research has shown that IL-4 is involved in suppression of immunity against cancers, such that blockade of IL-4 leads to enhanced anti-tumor immunity, reduced tumor progression, and increased tumor-specific cytotoxic T lymphocytes [18]. On the other hand, IFNγ induces many signals in T cells to efficiently enable T cell function, while the inhibition of IFNγ signaling pathways in T cells reduces T cell responses and permits tumor growth and persistence. IFNγ signaling also enhances tumor eradication by blocking the functions of some suppressive immune cells in the tumors, including Tregs [19].

In order to keep T-cell tolerance to self-antigens, Treg cells are vital. Treg cells, which are CD4^+^CD25^+^FoxP3^+^ cells, belong to the family of CD4^+^ T cells. These cells reduce immunity to tumor-related antigens by suppressing the natural killer (NK) cell-mediated cytotoxicity as well as inhibiting the interferon (IFN)-γ secretion by immune cells [20]. Thus, these cells impair effective anti-tumor immune response and are the leading obstacle in successful immunotherapy [21]. Accordingly, it has been revealed that the percentage of CD4^+^CD25^+^Foxp3^+^ Treg cells is elevated in the tumor micro-environment of the patient with metastatic breast cancer [22].

JAK2/STAT3 signaling pathway, which is extremely related to cancer initiation, metastasis, progression, chemoresistance, and immune evasion, is assumed to be a central mediator of tumor-related immune suppression [23–25]. STAT3 is critical in the molecular pathway required for FOXP3 expression and therefore is involved in Treg phenotype and function maintenance [26]. It has been revealed that STAT3 inhibition using small interfering RNA molecules, abrogated Foxp3 expression and suppressive functions in naturally differentiated CD4^+^CD25^+^ T lymphocytes, signifying a direct role for STAT3 in Treg function and phenotype [26]. Growing evidence shows that targeting STAT3 and its associated pathways can be used to change the immunologic microenvironment of tumors in order to support cancer immunotherapies [27–29]. In continuation of our research on the effects of inhibition of JAK2/STAT3 signaling pathway in breast cancer tumor cells [30, 31], the aim of this study was to investigate whether CPT, naringenin, and their combination could modify immune response, immune cell proliferation, and cytokine production. We also aimed to explore the involvement of JAK2/STAT3 signaling pathway and if treatment with CPT and naringenin could eventually lead to suppression of tumor growth in a mouse model of breast cancer.

## Materials and methods

### Mice

Inbred female Balb/c mice with the age of 6–8 weeks, weighing approximately 18 to 20 g, were purchased from Pasture Institute of Iran (Tehran, Iran). Animals were maintained for one week under standard conditions (temperature: 24 ± 1 °C, relative humidity: 40%–80%), prior to experimentation and had free access to regular chow diet and purified water. The mice were kept in a specific-pathogen-free (SPF) facility under barrier conditions. Before processing, the animals undertook anesthesia by intraperitoneal injection of 80 mg/kg ketamine and 8 mg/kg xylazine. Euthanasia of the animals was performed by cervical dislocation under general anesthesia. All animals were treated in compliance with the guide for the care and use of laboratory animals [32]. The study was carried out in compliance with the ARRIVE guidelines [33], and it was approved by Ethics Committee of the Shahid Beheshti University of Medical Sciences.

### Spontaneous mouse mammary tumor (SMMT) production and treatment

Explants of SMMT, which is invasive ductal carcinoma, developed spontaneously in female Balb/c mice [34], were used for tumor formation in mice, as we formerly described [35]. Briefly, tumor tissue was isolated from the breast of cancer-bearing Balb/c mice and divided into pieces with dimensions of less than 0.5 cm^3^ by scalpel. Subcutaneous transplantation of each piece was performed into a syngenic female Balb/c mouse. After 19 days, when the size of the tumor got to 1500 mm^3^, treatment with the substances was started. Mice were categorized into four groups (5 mice in each group) that were treated with naringenin either individually (50 mg/kg/day) or in combination with CPT (20 mg/kg/day). A group of mice received CPT alone (20 mg/kg/day) for comparison. Naringenin and CPT were purchased from Sigma-Aldrich (Germany) and both compounds were dissolved in dimethyl sulfoxide (DMSO) to prepare the stock. In order to treat the animals, the primary stock was further diluted with saline and injected intraperitoneally, so that the amount of DMSO was less than 1 ml/kg; a concentration that has been shown to be nontoxic [36]. The mice in control group were treated with saline containing the corresponding concentration of DMSO. A digital caliper was used to measure the volume of each tumor. The following formula was used for calculating the tumor size:

V = 1/ 2 × LW^2^ (V: volume, L: length and W: width).

### Antigen preparation

After tumors reached a volume of about 3000 mm^3^, tumor tissues were removed from the mice and mixed in RIPA buffer containing phenylmethylsulfonyl fluoride (PMSF) (1.0 mM concentration), as the protease inhibitor. Tumor cell lysate was prepared by successive freeze/thawing followed by passing through 150 µm stainless steel mesh, and subsequently sonicating for 30 s by 4 W power with 20 s incubation intervals. Then the extract was filtered and the concentration of its total protein was determined using the Bradford method. The lyaste was stored frozen at -70 ^◦^C for the subsequent experiments.

### Delayed type hypersensitivity (DTH)

The mice were primed by subcutaneous injection of 1 × 10^9^ (0.1 mL) mouse red blood cells of sheep (sRBC) in the back. sRBCs were washed three times, and diluted in 0.9% sodium chloride (NaCl) solution. In order to evaluate DTH, the sensitized animals were challenged by subcutaneous injection of sRBC (1 × 10^9^) on the left hind footpad, 5 days after priming. The intensification of the footpad thickness was quantitated before injection and after 24, 48, and 72 h, by a digital caliper, and the footpad swelling was compared in those receiving 0.9% NaCl solution or the antigen. The sensitized mice were categorized into five groups as follows: 1- control group receiving saline; 2- naringenin with the dose of 20 mg/kg/day; 3- naringenin with the dose of 50 mg/kg/day; 4- CPT (20 mg/kg/day); 5- CPT combined with naringenin, both with the dose of 20 mg/kg/day. The percent increase in footpad indicating DTH was evaluated in each group and compared with the control group.

### Cell proliferation assay

Experimental mice were sacrificed and the spleens were separated. Isolation of splenocytes was performed in sterile RPMI 1640 by the needle perfusion method and the erythrocytes were removed by lysing in the Ammonium-Chloride-Potassium (ACK) lysis buffer, containing NH_4_Cl (0.15 mM), Na_2_EDTA (0.1 mM) and KHCO_3_ (10 mM), at room temperature. After washing the cells, they were resuspended in RPMI 1640, including fetal bovine serum (FBS) (10%) and penicillin/streptomycin antibiotic mixture (50 I.U./mL penicillin and 50 (μg/mL) streptomycin). Cells were counted with a hemocytometer under invert microscope and were seeded in 96-well plates at the density of 5 × 10^5^ cells/well in 200 µl of phenol red-free RPMI 1640 supplemented with 10% heat-inactivated FBS, penicillin/streptomycin and 25 mM HEPES. The splenocytes were stimulated with 25 µg/ml of the tumor antigen that was previously prepared (explained above) as the test group. The cells were incubated at 37 ◦C for 36 h in a humidified atmosphere containing 5% CO_2_. Subsequently, cells were treated with naringenin alone (50 mg/kg/day) (Nar), CPT alone (20 mg/kg/day), or naringenin (20 mg/kg/day) + CPT (20 mg/kg/day) (Nar + CPT) compared with the untreated control group.

Cell proliferation was assessed with Bromodeoxyuridine (BrdU) method, using the cell proliferation BrdU assay kit (Roche Diagnostic GmbH, Mannheim, Germany), as instructed by the manufacturer. Briefly, 20 μl/well BrdU labeling solution was added to the cells and incubated for 24 h at 37 °C. Then, the microplates were centrifuged at 300 × g for 10 min, followed by aspirating the medium and drying the cells. After cell fixation, 100 μl/well anti-BrdU-POD were added to each well. Plates were incubated for 90 min at room temperature and after washing the wells, 100 μl substrate solution was poured into each well. After color development, the absorbance was measured in a plate reader (BioTek, Synergy HTX, USA), at 370 nm (with 492 nm reference wavelength).

### Measurement of cytokines profile

Splenic mononuclear cells (MNCs) were isolated from the spleens of treated and untreated animals by density gradient centrifugation, using Ficoll Hypaque (15 min,700 × g) at 20^◦^C. Cells were cultured in 24-well plates, at the condition mentioned above. Twenty microliters of purified tumor antigen was added to each well and after 72 h, supernatants were collected by centrifugation of plates for 10 min (Eppendorf). The supernatants were maintained frozen at -70 °C until future use. The concentrations of IFN-γ and IL-4 were measured using enzyme-linked immunosorbent assay (ELISA) (R&D DuoSet ELISA Development kit, Germany), following the manufacturer's protocols.

### Flow cytometric evaluation of the subpopulation T-lymphocytes

The removed spleens of the animals and the tumor tissues were separately cut into small pieces, rinsed with PBS, and minced with scalpel. In order to make cell suspensions, this mixture was passed through a 150-µm stainless steel mesh. The resulting cells were washed twice with phosphate-buffered saline (PBS) and mixed with PBS buffer containing heat-inactivated FBS (1%) (GIBCO, Germany), 0.1% sodium azide (Sigma, Germany), and 2 mM EDTA (Sigma, Germany) together with monoclonal antibodies against CD4, CD25 and Foxp3 surface antigens, using mouse Treg Detection Kit (CD4/CD25/FoxP3) (Miltenyi Biotec, USA), following manufacturer’s protocol. Samples were incubated with antibodies for 45 min at 4 ^◦^C followed by washing in buffer and fixing with paraformaldehyde (2%). Subsequently, flow cytometry assay was carried out by an EPICS flow cytometer (Coulter). The gate was adjusted on the lymphoid areas of forward and side scatter areas, and the cells that were double stained were analyzed.

### Western blot analysis

Tumor tissues were separated and cell suspensions were prepared as explained above. Tumor infiltrating lymphocytes were isolated from the cell suspension with Ficoll-Hypaque (Lympholyte-H; Cedarlane Laboratories, Ontario, Canada). After centrifugation at 1025 g for 20 min at 20ºC, the layer of mononuclear cells were transferred to a separate tube and washed twice with complete RPMI medium. The isolated lymphocytes were lysed with RIPA buffer containing PMSF as the protease inhibitor and the protein content of the cell lysate was assessed utilizing bicinchoninic acid assay kit (Thermo Fisher Scientific, UK). Equal amounts of total protein (40 µg total protein/lane) were loaded on 10% SDS-PAGE. After separation of proteins, they were transferred onto polyvinylidene fluoride (PVDF) membranes (Millipore, USA) via semi-dry blotting. The membranes were blocked with 5% non-fat milk at room temperature for 1 h. Subsequently, the membranes were incubated in the buffer solution containing the 1:1000 dilution of each of the primary antibodies against phosphorylated JAK2 (Cell Signaling, USA), phosphorylated STAT3 (Cell Signaling, USA), JAK2 (Cell Signaling, USA), STAT3 (Cell Signaling, USA), and β-actin (Sigma-Aldrich, Germany). Horseradish peroxidase (HRP)-conjugated anti-mouse IgG antibody (Santa Cruz Biotechnology, UK) was used as the secondary antibody and the succeeding visualization was done with SuperSignal chemiluminescent kit (Thermo Fisher Scientific, UK).

### Real-time PCR

Total RNA was isolated by RNeasy mini kit (Qiagen, Germany), as stated by the manufacturer. The complementary DNA (cDNA) was synthesized by reverse transcriptase enzyme using 1 µg/ml RNA, oligo-d(T)15 primer (Roche Applied Sciences), and molony murine leukemia virus reverse transcriptase (MMLV; Gibco). Target genes were amplified and analyzed by real-time PCR using SYBR-green and specific primers (Table 1).

**Table 1:** The sequences of the primers

Gene name	Forward primer	Reverse primer
Mcl-1	5'-AGAAAGCTGCATCGAACCAT-3	5'-CCAGCTCCTACTCCAGCAAC-3
Bcl-XL	5'-CTGAATCGGAGATGGAGACC-3	5'-TGGGATGTCAGGTCACTGAA-3
GAPDH	5'-ACCCACTCCTCCACCTTTGA-3	5'-CT GTTGCTGTAGCCAAATTCGT-3

### Statistical analysis

The results were analyzed as the mean ± standard deviations (SD) of the results, obtained from at least three separate experiments, each performed as triplicate. Statistical analysis was carried out by one-way analysis of variance (ANOVA) with Dunnett's post hoc test for multiple comparisons as well as two-tailed Student’s t-test in SPSS software (version 16). Results were considered as statistically significant when the *P* value of < 0.05 was obtained.

## Results

### Effect of Naringenin and CPT on tumor volume

**Fig. 1:**
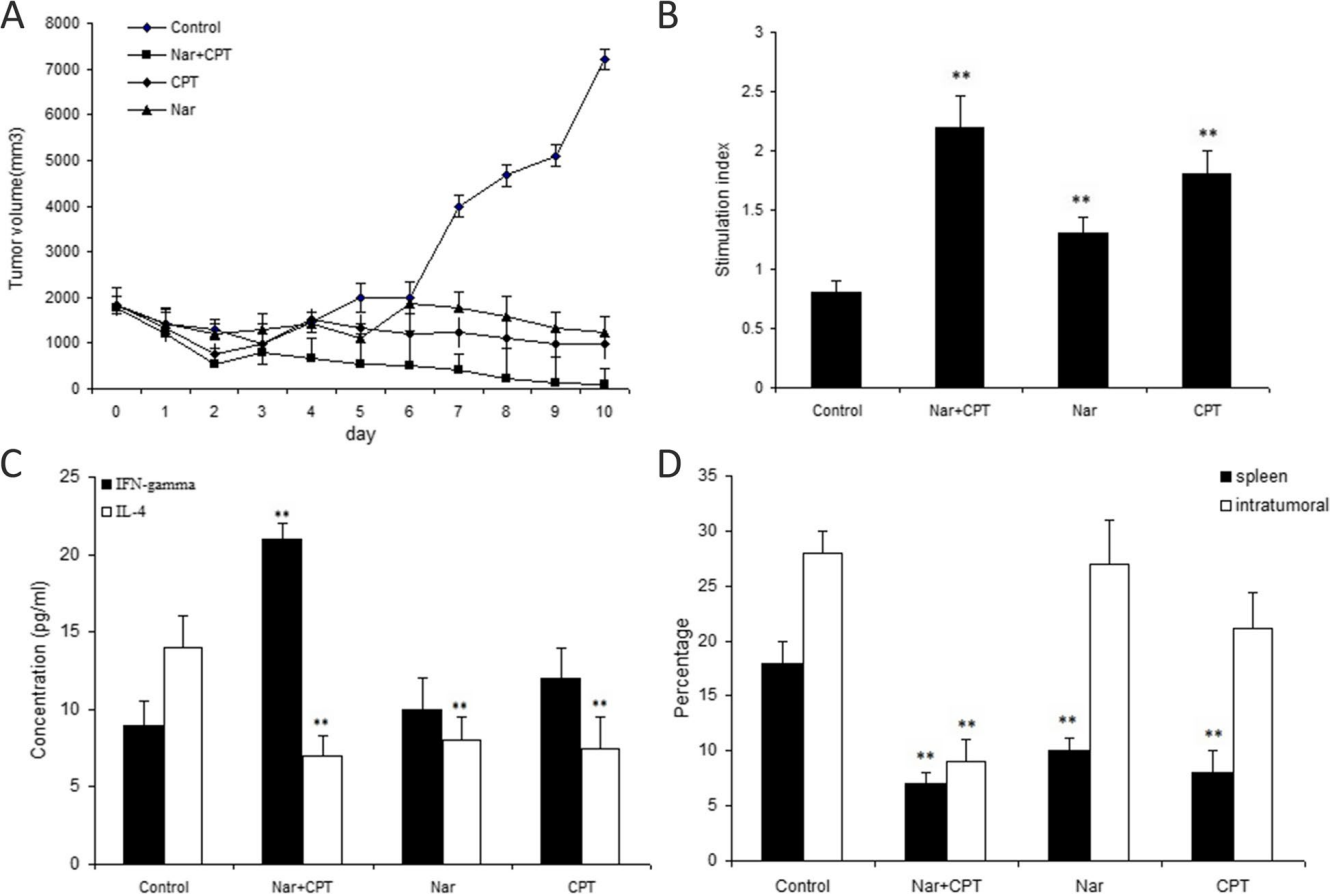
**A** The tumor volume (Mean ± SD) in the treated and untreated control group (5 mice/group). Treated group consisted of mice (5 mice in each group) receiving daily intraperitoneal injections of naringenin (50 mg/kg/day) or CPT (20 mg/Kg/day) or their combination (naringenin + CPT) for 10 consecutive days. **B** Proliferation assay of splenocytes treated with naringenin alone (50 mg/kg/day) (Nar), CPT (20 mg/Kg/day), or naringenin (20 mg/kg/day) + CPT (20 mg/kg/day) (Nar + CPT) compared with the untreated control group (receiving DMSO as the solvent). **C** Levels of IFN-γ and IL-4 secreted from splenocytes stimulated by tumor antigen in animals treated with naringenin (Nar) alone or combined with CPT (Nar + CPT). **D** Percentage of intra-tumoral and splenic CD4^+^ CD25^+^ Foxp3^+^  T cells in animals treated with naringenin (Nar), either individually (50 mg/kg/day) or in combination with CPT (both with the concentration of 20 mg/kg/day). *N* = 5 in each group. * *P* < 0.05 and ** *P* < 0.01 represents significant differences in comparison with control values

As shown in Fig. 1A, intraperitoneally injection of naringenin, either individually or in combination with CPT, resulted in a remarkable tumor regression in comparison with the control group. Combination of CPT and naringenin caused a more prominent decline in the rate of tumor growth, compared with those receiving only naringenin or CPT individually; an effect that became significantly different after day 7.

### Effect of naringenin and CPT on DTH response

The results of naringenin and CPT treatment on immune response is presented in Table 2. DTH responses were significantly different from control group in mice receiving naringenin with the dose of 50 mg/Kg/day (group 3) and those that were treated with the combination of naringenin and CPT (group 5) (respectively 1.8 fold and 2.4 fold higher than control group after 24 h). The combinational naringenin + CPT regimen resulted in the most prominent DTH response (*P* < 0.05).

**Table 2:** The effect of naringenin and CPT on DTH responses in Balb/c mice against red blood cells of sheep (sRBC)

	treatment	% of footpad increase (DTH)		
		24 h	48 h	72 h
Group 1	sRBC + saline	45 ± 4.2	42 ± 8.2	35 ± 5.8
Group 2	sRBC + Naringenin (20 mg/Kg/day)	70 ± 8.9	65 ± 7.8	60 ± 6.3
Group 3	sRBC + Naringenin (50 mg/Kg/day)	93 ± 7.7^a^	76 ± 8.4	54 ± 2.3
Group 4	sRBC + CPT (20 mg/Kg/day)	75 ± 8.4	61 ± 8.4	59 ± 7.3
Group 5	sRBC + Naringenin (20 mg/Kg/day) + CPT (20 mg/Kg/day)	108 ± 5.4^a^	96 ± 6.4^a^	72 ± 5.3

Results are presented as mean ± S.E.M. (standard error of the mean)

*DTH* Delay type hypersensitivity, *CPT* Cryptotanshinone

^a^Significant difference (*P* < 0.05) in comparison with group 1 (control)

### Effect of Naringenin and CPT on cell proliferation

The proliferation of splenocytes were investigated after treatment with naringenin (50 mg/Kg/day) or its combination with CPT (20 mg/Kg/day). The results are presented in Fig. 1B and reflect the mean values of triplicates after stimulation with specific tumor antigen. Naringenin significantly induced cell proliferation, compared with the control cells (1.25 fold increase compared to control cells, *P* < 0.05). When used together with CPT, naringenin caused approximately 2.2 fold increase in cell proliferation which was significantly higher than control cells (*P* < 0.01).

### The effect of naringenin and CPT on IFN-γ and IL-4 production

The levels of IL-4 and IFN-γ, secreted from the splenocytes of treated and untreated tumor-bearing animals, are presented in Fig. 1C. Treatment with naringenin significantly reduced IL-4 levels compared with control and caused about 35% reduction in IL-4 levels. However, when naringenin and CPT were co-administered, IL-4 level was further decreased to half the level of that in control cells. Furthermore, our data demonstrated a significant upsurge in the production of IFN-γ by splenocytes of the mice that were treated by both naringenin and CPT (2.3 fold compared to control, *P* < 0.01). Although the levels of IFN-γ was approximately 1.2 fold increased in response to naringenin, treatment with naringenin alone did not have any significant effect on IFN-γ production compared to control, pointing out that here the CPT was the effective agent on IFN-γ production and secretion.

### Splenic and intra-tumor CD4^+^CD25^+^Foxp3^+^ regulatory T cells

Data revealed that frequencies of intra-tumoral and splenic CD4^+^CD25^+^Foxp3^+^ T cells were significantly lower than the control group in animals that were administered with both naringenin and CPT-treated animals (32.0% and 38.8% compared to control, respectively(P ˂0.01) (Fig. 1D). The frequencies of intra-tumor CD4^+^CD25^+^Foxp3^+^ T cells in animals treated only with naringenin did not show any significant difference; showing that CPT but not naringenin was able to modulate intratunoral Tregs. However, the number of splenic CD4^+^CD25^+^Foxp3^+^ T cells were significantly lower in naringenin-treated animals (55.5% compared to control) as well as combination of naringenin and CPT (Fig. 1D).

### Combined treatment with naringenin and CPT suppressed JAK2/STAT3 signaling pathway

**Fig. 2:**
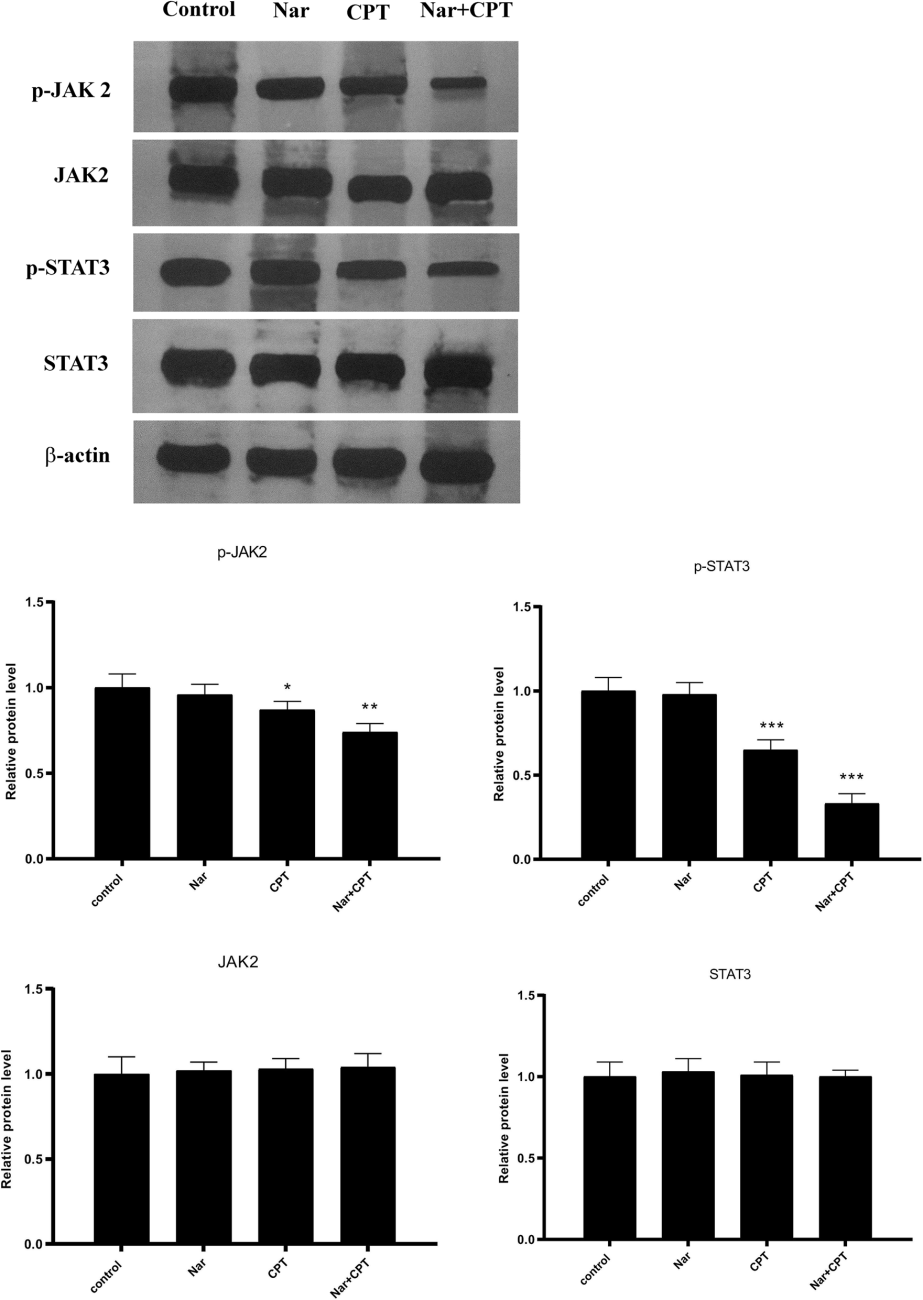
Phosphorylation status of JAK2 (p-JAK2) and STAT3 (p-STAT3) together with their total protein level in tumor infiltrating immune cells, evaluated by Western blotting after administration of naringenin (50 mg/Kg/day) (Nar) and CPT (20 mg/kg/day) either individually or together (both at the concentration of 20 mg/kg/day). The blots were visualized on the X-ray film after processing of the blots with enhanced chemiluminescent (ECL) reagent. The original images are presented in supplementary file with the edges of the blot images visible (the blots were cut prior to hybridization). The results of the densitometry of the bands are presented in the graphs. Protein level of β-actin was used for normalizing the results. * *P* < 0.05, **P < 0.01, *P* < 0.001, compared to control. #*P* < 0.05 compared to CPT

As shown in Fig. 2, individual treatment with naringenin was not able to decrease JAK2 and STAT3 phosphorylation. On the other hand, CPT which is a well-known inhibitor of STAT3, caused a significant decline in the phosphorylation of both JAK2 and STAT3. Interestingly, the addition of naringenin to CPT augmented the inhibitory effect of CPT, and further declined the phosphorylation of both JAK2 and STAT3. None of the compounds had any particular effect on total protein levels of JAK2 and STAT3, as well as β-actin protein expression level.

**Fig. 3:**
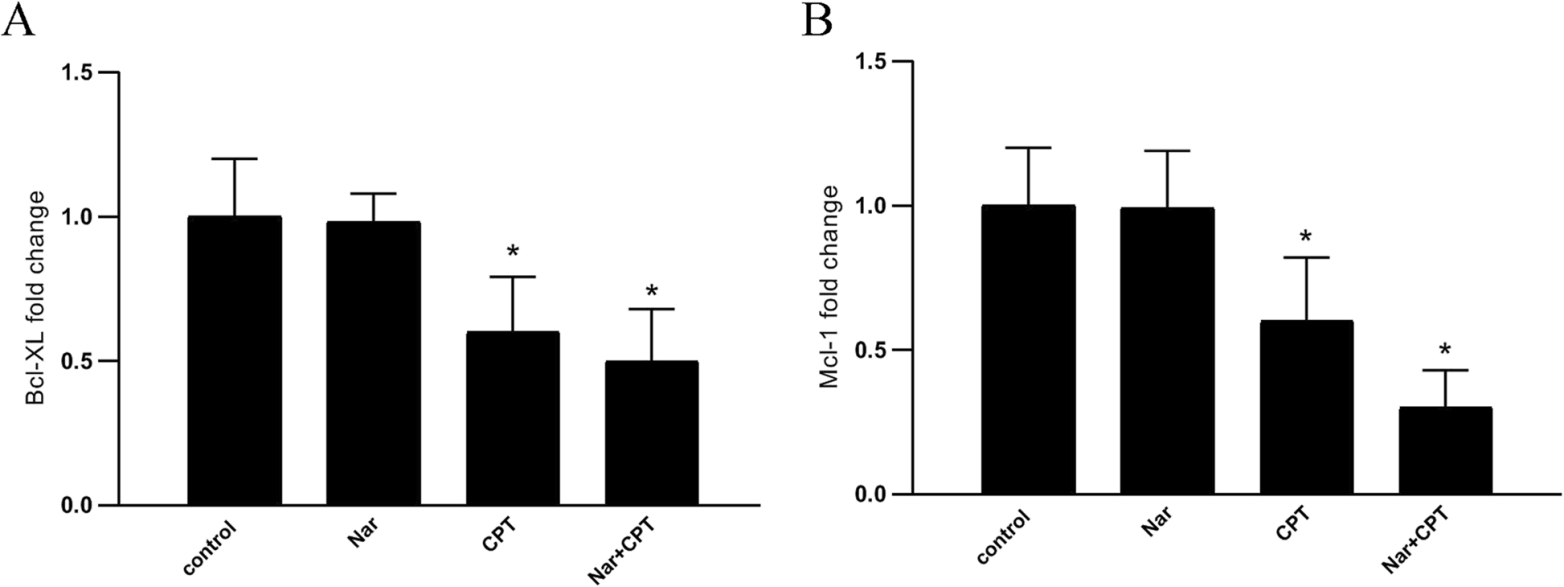
The expression of (**A**) *Bcl-XL* and (**B**) *Mcl-1* genes, after administration of naringenin (50 mg/kg/day) (Nar) and CPT (20 mg/kg/day) either individually or together (both at the concentration of 20 mg/kg/day). The expression of genes were measured by real-time PCR and analyzed using ΔΔCt method. * *P* < 0.05, **P < 0.01, *P* < 0.001, compared to control

Bcl-2 and Mcl-1 have long been recognized as the direct targets of STAT3. Thus, in order to confirm the down-regulation of STAT3 by naringenin and CPT, the expression of both *Bcl-XL* and *Mcl-1* genes were investigated. As it is shown in Fig. 3, although naringenin alone was not able to change the expression levels of the studied genes, both CPT and its combination with naringenin were able to reduce the expression of *Bcl-XL* and *Mcl-1* genes (Fig. 3).

## Discussion

Cancer immunotherapy, the immune system’s artificial stimulation, as well as the use of natural products have been proposed as efficient methods in the treatment of different cancers [13, 37]. In this study, we sought to reveal anti-tumor and immunomodulatory properties for CPT and naringenin and their combination, in order to establish their potential as adjuvant therapy in breast cancer. We first showed that naringenin was able to significantly decrease tumor size, an effect that was potentiated with the addition of CPT. Consistently, several previous articles have reported anticancer activities for CPT, as a specific natural STAT3 inhibitor [38], as well as naringenin, a well-known natural flavonoid, against different types of cancers [39–44].

Recently, numerous immune-based therapies have been established to be beneficial for the eradication of tumors in animal models. Primary immune organs, including the spleen and thymus, play vital roles in controlling the anti-tumor immunity [45]. The spleen is the principal peripheral lymphoid organ in the body and is the location of B and T cells. Lymphocyte activity and proliferation in the spleen represent its immune ability and the cellular immune function of the body [46]. Here we showed increased proliferation of splenocytes in response to treatment with naringenin and its combination with CPT, suggesting that these two compounds influence the immune system. A significant elevation was also observed in the DTH response especially in the naringenin + CPT group in comparison with the control group, further confirming their immunomodulatory properties. Naingenin has been previously reported to be able to exert immunomodulatory effects. In mouse model of invasive melanoma, naringenin promoted NK cell differentiation, maturation, and cytotoxicity against cancer, and its combination with asiatic acid produced an additive effect [47]. Other natural substances such as curcumin and resveratrol have also shown immunomodulatory properties [48].

Cancer is accompanied by chronic inflammation in which immune cells enhance rather than suppress tumor growth. Thus, changing the type of inflammation to the one that causes tissue rejection, is an attractive method to eradicate tumors by immune effector cells. Th cells range from those that can dampen the immune response (such as Foxp3^+^ Treg), to those that can cause tumor destruction (such as IFN-γ-secreting Th1). Thus, shifting the immune response towards the latter and the induction of tumor antigen specific Th1 immunity may be of benefit in the treatment of cancer patients.

Our results demonstrated for the first time that naringenin led to a change in Th1/Th2 balance towards Th1-dominant immunity; an effect that was potentiated by coadministration of CPT. It has been previously reported that the frequency of CD4^+^CD25^+^Foxp3^+^ Treg cells is amplified in the tumor micro-environment and peripheral blood mononuclear cells, indicating that the enhancement of Treg cells is a widespread problem [22]. Thus, decreased percentage of CD4^+^CD25^+^Foxp3^+^ in both spleen and breast tumor tissue by naringenin and CPT might be of significant benefit for breast cancer immunotherapy.

Treatment with naringenin, either alone or combined with CPT, also resulted in a significantly reduced production of IL-4, the most important Th2 cytokine that promotes the proliferation and survival of several cancer cells [49]. Additionally, coadministration of naringenin and CPT increased production of IFN-γ, an important effector cytokine of anti-tumor immunity [50]. In line with our findings, naringenin has been previously introduced as an effective substance in reducing the production of the pro-inflammatory factor TNF-α and attenuating the levels of IL-1β and IL-6 in human cultured macrophages [51]. Another study also described enhanced antitumor activity of the T cells after treatment of mouse model of breast cancer with naringenin, with an increased proportion of IFN-γ and IL-2 expressing T cells [52]. Additionally, Naringenin has been shown to be able to improve the immunosuppressive environment of lung tumor by reducing transforming growth factor-beta1 production and diminishing the number of regulatory T cells [53].

Previous studies have provided evidence that the anticancer activity of naringenin and CPT occurs via inhibition of STAT3 signaling pathway in pancreatic cancer cells and vascular endothelial cells [54, 55]. The results of our study revealed that CPT is capable of suppressing JAK2 and STAT3 phosphorylation in breast cancer cells and its combination with naringenin exerts as additive effect on JAK/STAT pathway. In line with our findings, CPT has been shown to inhibit the phosphorylation of STAT3 at Tyr705 in a dose-dependent manner, without noticeable effects on the total level of STAT3 in pancreatic cancer cells [55]. The short term effect of CPT on STAT3 phosphorylation has suggested a direct effect of this substance [55]. The same effect has also been observed for CPT in prostate cancer cells [38]. Consistently, naringenin has also been introduced as an effective inhibitor of STAT3 phosphorylation in vascular endothelial cells [56]. Although we did not observe a significant change in STAT3 and JAK2 phosphorylation in response to naringenin, the augmented suppression of STAT3 and JAK2 phosphorylation after co-administration of naringenin and CPT, highlights that these two substances act in the same direction and therefore they form a suitable combination. Consistently the effect of naringenin on some other studied parameters was modest or nonsignificant; nevertheless, the same concentration could potentiate CPT to exert higher effect.

A previous study by Pallandre et al. demonstrated that suppression of STAT3, using small interfering RNA molecules, leads to repressed Foxp3 expression and functions of differentiated CD4^+^CD25^+^ T lymphocytes [26]. Moreover this study reported that STAT3 inhibition in CD4^+^ lymphocytes improved the anti-tumor immunity conferred by a lymphocyte adoptive transfer [26]. Accordingly, the decline in the frequencies of CD4^+^CD25^+^Foxp3^+^ T cells in the mice treated with the combination of naringenin and CPT might be attributed to the suppression of STAT3 signaling pathway. STAT3 is a well-known regulator of Bcl-2 family genes including Bcl-xL [57]. Mcl-1 which is another member of Bcl-2 family, is an imperative anti-apoptotic protein in the development of multiple cell types including T lymphocytes [58]. Downregulation of Bcl-xL and Mcl-1 further confirms the manipulation of STAT3 pathway by the combined treatment of naringenin and CPT.

The limitation of our study is that we could not investigate molecular interaction between the studied compounds and JAK/STAT pathway and therefore we cannot conclude a direct effect on this pathway. Another limitation is that we could not expand our study to evaluate complete profile of proinflammatory and anti-inflammatory cytokines which is suggested for future studies.

Current cancer treatments have low efficiency and exhibit toxicities that are usually harmful. Numerous evidences support that agents derived from plants have more pharmacological safety and can be useful not only for the prevention of cancer but also for its treatment. The results of this study show that naringenin and CPT exert immunomodulatory effects and have the molecular targets that are similar to the therapies currently used to treat cancer. Thus, these agents can be used as adjuvants to current chemotherapy agents to enhance therapeutic effects and minimize therapy-induced toxicity.

## Conclusion

The present study demonstrated an immunomodulatory and antitumor effect for naringenin and its combination with CPT in an animal model of breast cancer. Attenuation of JAK/STAT pathway in breast cancer, the shift in immune response towards Th1, and the consequent changes in the secretion of the relevant cytokines may be the mechanisms by which naringenin and CPT effectively suppress breast cancer. Thus, naringenin and CPT may be considered as a novel combination for supplementation in breast cancer immunotherapy trials.

## Supplementary Information

**Additional file 1.**

## Data Availability

The datasets used and/or analysed during the current study are available from the corresponding author on reasonable request.
