# Supplementary material for: Naringenin and cryptotanshinone shift the immune response towards Th1 and modulate T regulatory cells via JAK2/STAT3 pathway in breast cancer
Source: BMC Complement Med Ther. 2022 May 23;22:145. doi: 10.1186/s12906-022-03625-x (PMC9125892; doi:10.1186/s12906-022-03625-x)
Supplement: Supplementary file 1 — Additional file 1. [file 12906_2022_3625_MOESM1_ESM.docx]

**Supplementary figure**

The original images of the blots of the indicated proteins, visualized by enhanced chmiluminescent (ECL) substrate on X-ray film (The films were cut prior to developing the bands images).


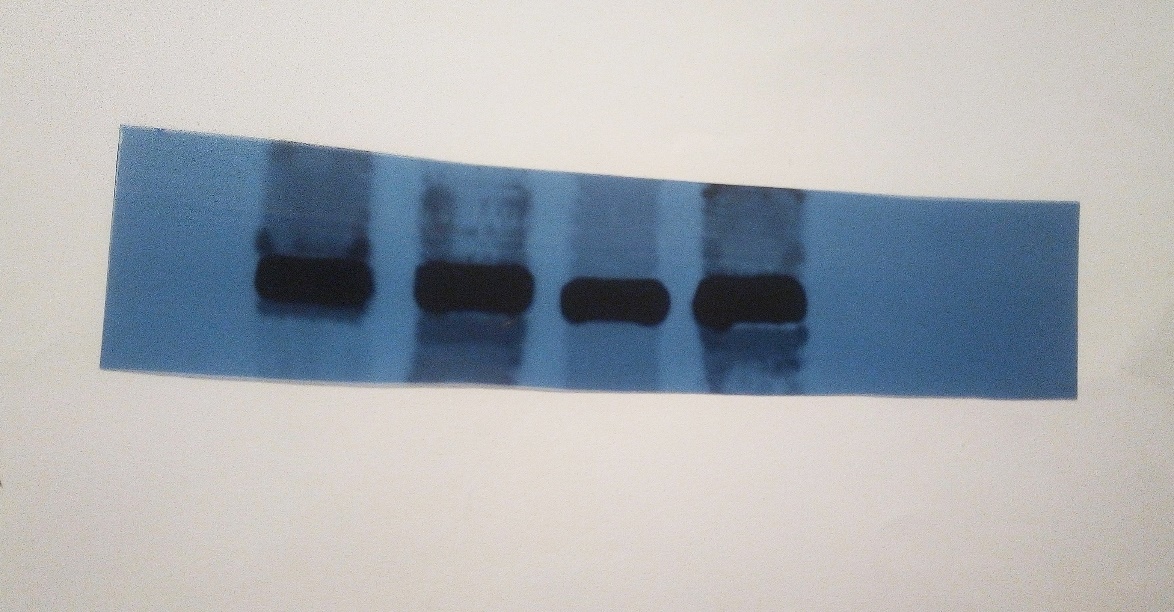

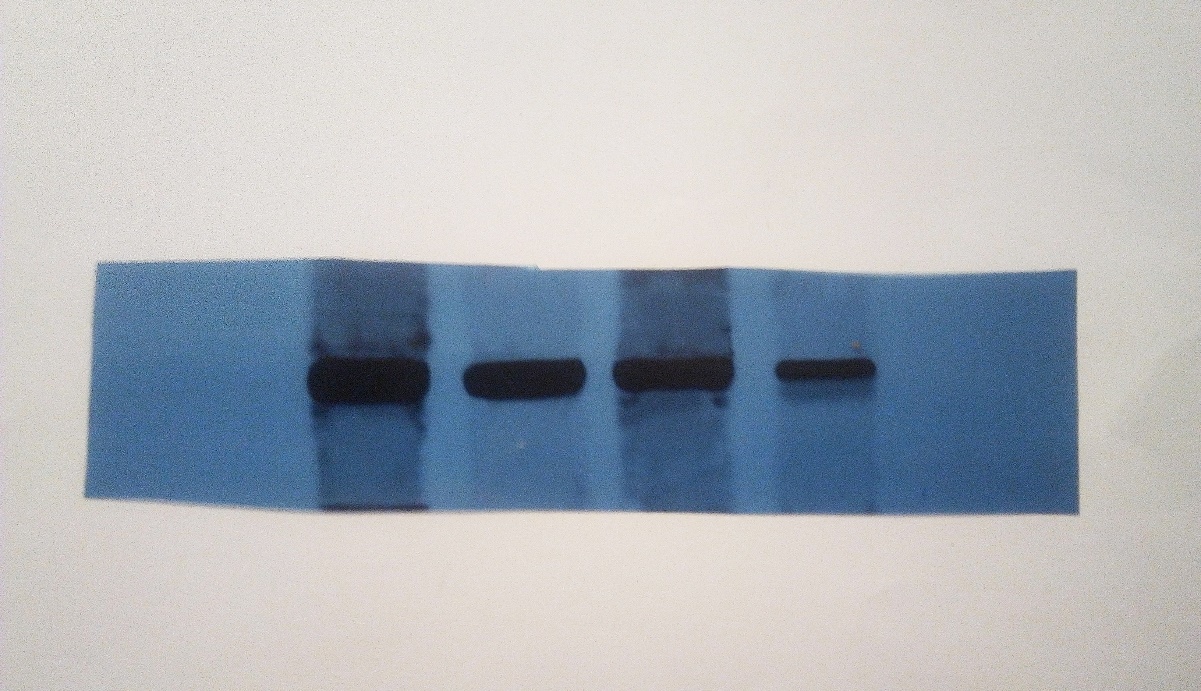


**JAK-2**

**p-JAK-2**

**130 kDa**

**130 kDa**

**p-STAT3**


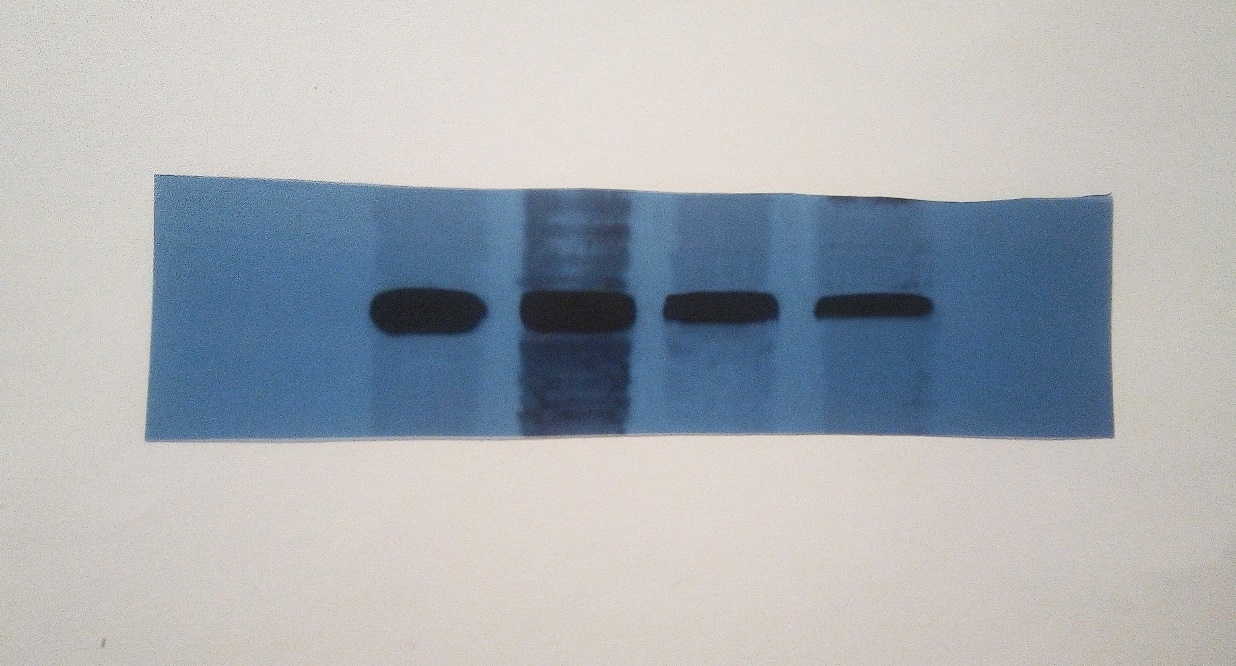


**100 kDa**

**70 kDa**

**STAT3**


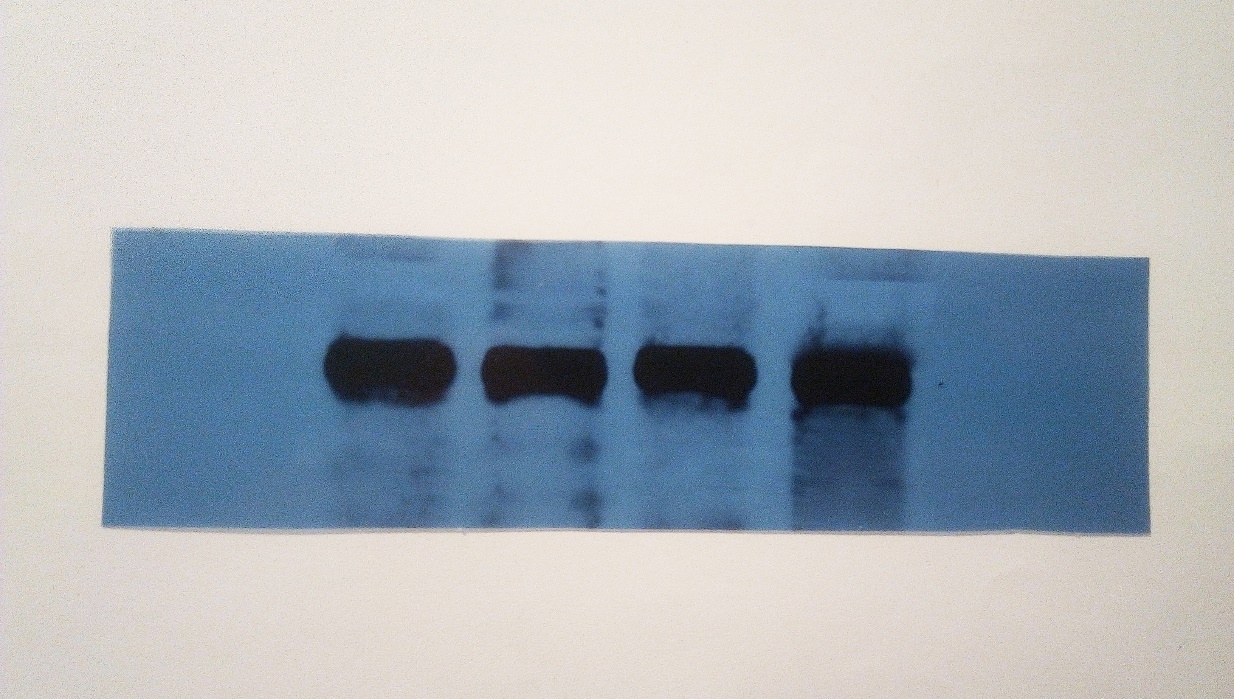


**70 kDa**

**100 kDa**

**β-Actin**


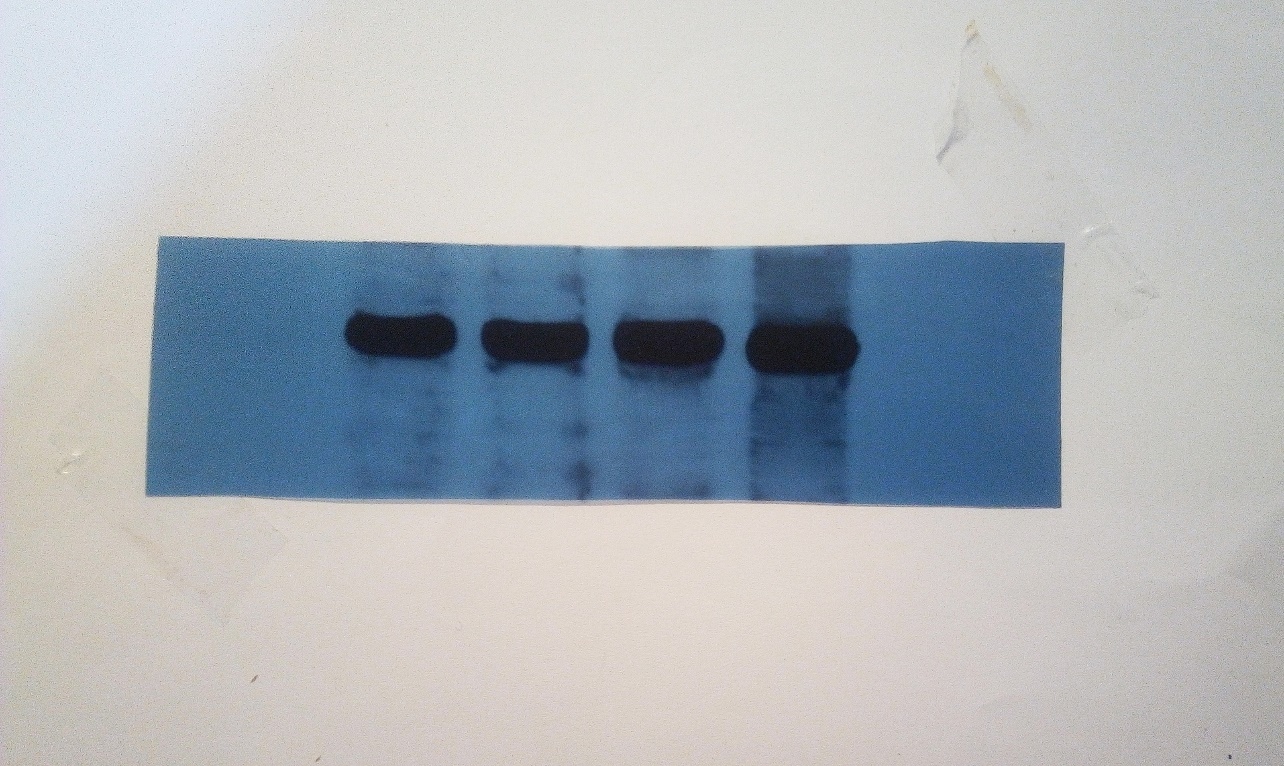


**40 kDa**

**55 kDa**
